# Controlling Adsorption of Diblock Copolymer Nanoparticles onto an Aldehyde-Functionalized Hydrophilic Polymer Brush via pH Modulation

**DOI:** 10.1021/acs.langmuir.3c03392

**Published:** 2024-02-06

**Authors:** Samuel Astier, Edwin C. Johnson, Oleta Norvilaite, Spyridon Varlas, Emma E. Brotherton, George Sanderson, Graham J. Leggett, Steven P. Armes

**Affiliations:** † Department of Chemistry, The University of Sheffield, Dainton Building, Brook Hill, Sheffield, South Yorkshire S3 7HF, U.K.; ‡ GEO Specialty Chemicals, Hythe, Southampton, Hampshire SO45 3ZG, U.K.

## Abstract

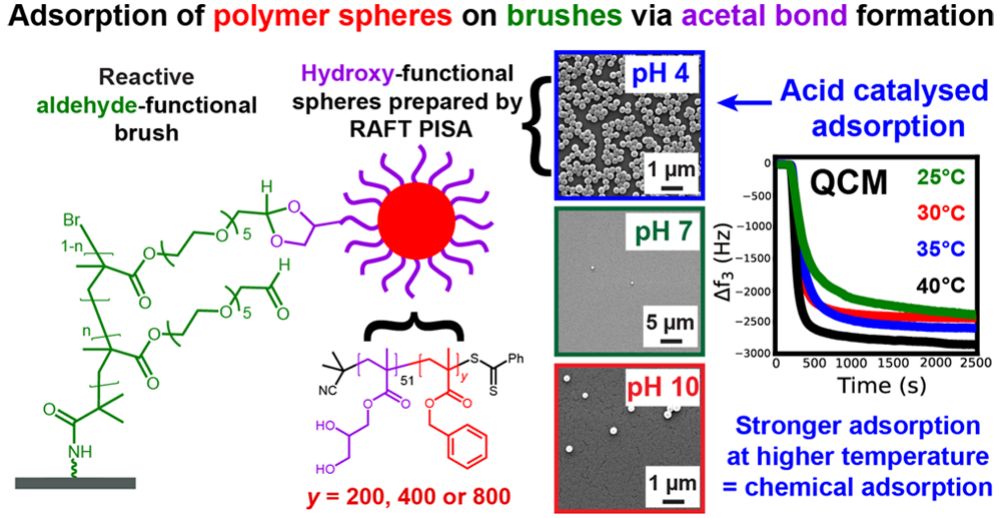

Sterically stabilized diblock copolymer nanoparticles with a well-defined spherical morphology and tunable diameter were prepared by RAFT aqueous emulsion polymerization of benzyl methacrylate at 70 °C. The steric stabilizer precursor used for these syntheses contained pendent *cis*-diol groups, which means that such nanoparticles can react with a suitable aldehyde-functional surface via acetal bond formation. This principle is examined herein by growing an aldehyde-functionalized polymer brush from a planar silicon wafer and studying the extent of nanoparticle adsorption onto this model substrate from aqueous solution at 25 °C using a quartz crystal microbalance (QCM). The adsorbed amount, Γ, depends on both the nanoparticle diameter and the solution pH, with minimal adsorption observed at pH 7 or 10 and substantial adsorption achieved at pH 4. Variable-temperature QCM studies provide strong evidence for chemical adsorption, while scanning electron microscopy images recorded for the nanoparticle-coated brush surface after drying indicate mean surface coverages of up to 62%. This fundamental study extends our understanding of the chemical adsorption of nanoparticles on soft substrates.

## Introduction

Understanding and controlling particle adsorption at interfaces is fundamental to commercial products such as paints^1^ and coatings,^2,3^ as well as for the preparation of colloidosomes,^4^ Pickering emulsions,^5^ liquid marbles,^6−9^ and long-lived foams.^10^ In principle, such adsorption may involve physical interactions such as electrostatic attraction, hydrogen bonding, van der Waals forces, or the formation of new bonds (i.e., chemical adsorption).

The quartz crystal microbalance (QCM) is a well-established analytical technique in surface science. It is extremely sensitive to changes in mass, *m*, owing to adsorbed species because this causes a reduction in the frequency of an oscillating quartz crystal, Δ*f*, which can be determined with high precision. Sauerbrey derived a simple relationship, which has become widely known as the Sauerbrey equation (eq 1).
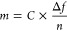
1

Here *C* is a sensitivity constant (expressed in units of mg m^–2^ Hz^–1^) and *n* is the overtone number.

Accordingly, QCM has been used to monitor the adsorption of a range of colloidal particles onto various model planar substrates, including gold,^11^ silica,^12^ cellulose,^13^ and stainless steel.^14,15^ Literature examples include the electrostatic adsorption of anionic silica nanoparticles onto a modified cationic substrate,^16^ the adsorption of gold nanoparticles for the design of biosensors,^17−20^ and the adsorption of charged diblock copolymer micelles onto oppositely charged surfaces.^21−26^ Under appropriate conditions, QCM-D (where D denotes dissipation) can also be employed to reveal structural information regarding the nature of the adsorbed material.^27,28^ However, most of these prior studies (i) have focused on physical adsorption rather than chemical adsorption and (ii) involve relatively hard (as opposed to soft^29^) substrates.

Over the past decade or so, polymerization-induced self-assembly (PISA) has become widely recognized as a powerful platform technology for the rational synthesis of a wide range of block copolymer nanoparticles.^30−38^ PISA involves the chain extension of a soluble block A in a suitable solvent using a monomer that forms an insoluble block B. Accordingly, in situ self-assembly occurs at some critical degree of polymerization (DP) for the second block to produce sterically stabilized nanoparticles, whereby the B block is located within the cores and the A block confers steric stabilization.^39,40^ Various research groups have developed highly efficient one-pot syntheses for many PISA formulations.^41−43^ Moreover, PISA can be conducted using a wide range of solvents (including either water or oil) and the overall particle diameter can be systematically varied over a wide range in the case of kinetically trapped spheres.^26,44−51^ This enables the convenient synthesis of libraries of sterically stabilized nanoparticles for model studies simply by adjusting the target DP for the insoluble core-forming block.^52−54^

For example, introducing epoxy groups into hydrophobic nanoparticles leads to their much stronger adsorption from mineral oil onto stainless steel.^15^ Enhanced adsorption from aqueous solution onto the same planar substrate was also achieved for suitably modified hydrophilic nanoparticles.^14^ PISA has also proved to be useful for establishing the essential design rules for nanoparticle occlusion within calcite crystals^55,56^ and for new nanoparticle dispersants to produce concentrated aqueous suspensions of micron-sized azoxystrobin crystals (a widely used broad-spectrum fungicide).^57,58^

Very recently, we demonstrated that nanoparticle interactions with a hydrophilic polymer brush can be mediated by dynamic covalent chemistry.^59^ More specifically, aldehyde-decorated nanoparticles can adsorb strongly onto a primary amine-functionalized brush via Schiff base chemistry (i.e., the formation of multiple imine bonds). Herein we report that an aqueous PISA formulation can be used to prepare *cis*-diol-functionalized spherical nanoparticles of tunable size. The effect of varying the solution pH on the extent of adsorption of such nanoparticles onto a hydrophilic aldehyde-functional planar brush is monitored using a QCM and scanning electron microscopy (SEM). Overall, our experimental data provide strong evidence for the formation of acetal bonds, rather than merely physical adsorption.

## Experimental Section

### Materials

Unless stated otherwise, all of the reagents were used as received. Glycerol monomethacrylate (GMA) and the *cis*-diol-capped oligo(ethylene glycol) monomethacrylate (GEO5MA) monomer were donated by GEO Specialty Chemicals (Hythe, UK) and used without further purification. Benzyl methacrylate (BzMA), 4,4′-azobis(4-cyanopentanoic acid) (ACVA; 99%), (3-aminopropyl)triethoxysilane (APTES; 99%), 2-bromoisobutyryl bromide (BiBB; 98%), sodium periodate (NaIO_4_; > 99%), and dichloromethane (DCM; > 99%) were purchased from Sigma-Aldrich (UK). 2-Cyano-2-propyldithiobenzoate (CPDB) was purchased from STREM Chemicals Ltd. (Cambridge, UK). *N*,*N*,*N*′,*N*″,*N*″-Pentamethyldiethylenetriamine (PMDETA; 98%), tetrahydrofuran (THF), ethanol, dimethylformamide (DMF), and hydrochloric acid (HCl, 37%) were purchased from Fisher Scientific (UK). Sodium hydroxide pellets (NaOH, 98%) were purchased from Alfa Aesar. Copper(II) chloride (CuCl_2_; 99%) was purchased from Acros Organics (UK). Deuterated dimethylformamide (*d*_7_-DMF) was purchased from Goss Scientific Instruments Ltd. (Cheshire, UK). Test-grade silicon wafers (100) were purchased from PI-KEM (Tamworth, UK). Deionized water was used for all experiments involving aqueous solutions.

### Characterization Techniques

#### ^1^H NMR Spectroscopy

Spectra were recorded in *d*_7_-DMF using a 400 MHz Bruker AVANCE-400 spectrometer at 298 K with 16 scans being averaged per spectrum.

#### Aqueous Electrophoresis

Zeta potentials for diblock copolymer nanoparticles were analyzed using a Malvern Zetasizer Nano ZS instrument equipped with a 4 mW He–Ne laser (λ = 633 nm) operating at a fixed scattering angle of 173°. Samples were diluted to 1% w/w using 1 mM KCl, with either dilute NaOH or HCl being used for pH adjustment as required. Zeta potentials were calculated from the Henry equation using the Smoluchowski approximation.

#### DMF Gel Permeation Chromatography

DMF gel permeation chromatography (GPC) was used to assess the molecular weight distribution of each (co)polymer. The instrument setup comprised two Agilent PL gel 5 μm Mixed-C columns and a guard column connected in series to an Agilent 1260 Infinity GPC system operating at 60 °C. The GPC eluent was high-performance liquid chromatography-grade DMF containing 10 mM LiBr at a flow rate of 1.0 mL min^–1^, the copolymer concentration was typically 1.0% w/w, and calibration was achieved using a series of ten near-monodisperse poly(methyl methacrylate) (PMMA) standards ranging from 1080 to 905,000 g mol^–1^. Chromatograms were analyzed using Agilent GPC/SEC software.

#### Dynamic Light Scattering

Dynamic light scattering (DLS) studies were performed using a Malvern Zetasizer Nano-ZS instrument equipped with a 4 mW He–Ne laser (λ = 633 nm) operating at a fixed scattering angle of 173°. Copolymer dispersions were diluted to 0.1% (w/w) using deionized water prior to light scattering studies at 25 °C, with 2 min being allowed for thermal equilibrium prior to each measurement. The hydrodynamic z-average particle diameter was calculated via the Stokes–Einstein equation.

#### Spectroscopic Ellipsometry

Measurements were performed in air at 20 °C on bare planar silicon wafers, initiator-functionalized silicon wafers, and polymer brush-functionalized silicon wafers using a J. A. Woollam M2000 V ellipsometer at a fixed angle of incidence of 75° normal to the sample surface. A wavelength range of 370–1000 nm was used to obtain two ellipsometry parameters (Ψ and Δ). Data analysis and modeling were performed using Woollam CompleteEASE software. The Ψ and Δ values were fitted using a two-layer model comprising a native oxide layer and a Cauchy layer to describe the polymer brush. Cauchy parameters were *A*_n_ = 1.482, *B*_n_ = 0.0056 μm^–2^, and *C*_n_ = 0. The ellipsometer setup allowed a relatively large area (approximately 0.5 × 1 cm) to be sampled; this corresponds to around 30% of the total area of each brush sample.

#### Transmission Electron Microscopy

Copper/palladium transmission electron microscopy (TEM) grids (Agar Scientific, UK) were coated in-house with a thin film of amorphous carbon. Grids were then subjected to a glow discharge for 30 s to create a hydrophilic surface. Each 0.1% w/w aqueous diblock copolymer dispersion was deposited as a 5.0 μL droplet onto a freshly treated grid for 1 min and then blotted with filter paper to remove excess solution. To stain the deposited nanoparticles, uranyl formate (5.0 μL of a 0.75% w/w aqueous solution) was placed on the sample-loaded grid for 20 s and then carefully blotted to remove excess stain. Each grid was then dried using a vacuum hose. Imaging was performed using a FEI Tecnai Spirit 2 instrument operating at 80 kV and equipped with an Orius SC1000B camera.

#### Quartz Crystal Microbalance

QCM sensors coated with a 50 nm silica overlayer (QSX 303, ∼ 5 MHz fundamental frequency) were purchased from Q-Sense (Sweden). Each sensor was cleaned according to the manufacturer’s instructions. This protocol involved (i) UV/O_3_ treatment for 15 min (BioForce UV/O_3_ cleaner, ∼ 9 mW cm^–2^, λ = 254 nm), (ii) exposure to 2% w/w sodium dodecyl sulfate solution for 30 min, (iii) copious rinsing with deionized water and drying under N_2_, and (iv) a final UV/O_3_ treatment for 15 min. The resulting substrates were functionalized with the ATRP initiator prior to surface-initiated polymerization to produce brush-coated substrates using the protocols given below.

QCM measurements were performed using an openQCM NEXT instrument (Novaetech S.r.l., Italy) equipped with a temperature-controlled cell connected to a Masterflex Digital Miniflex peristaltic pump (Cole-Parmer Instrument Company, UK). A rapid flow of ethanol (2 mL min^–1^) was used to wet the inner cell surface. Once all bubbles had been removed, water (either pH 4, 7, or 10) was flowed through the cell until the sensor frequency exhibited a drift of less than 1 Hz min^–1^. This typically occurred within 1 h of filling the cell. Once a stable signal was obtained, a 1% w/w aqueous dispersion of nanoparticles at the desired solution pH was passed through the cell at a flow rate of 0.1 mL min^–1^ (minimum flow volume = 3 mL). Once any signal drift had abated, water at the same pH was passed through the cell at the same flow rate, and the experiment was terminated after returning to minimal signal drift. Most QCM experiments were performed at 25 °C but some measurements were also conducted at 30, 35, or 40 °C.

The adsorbed amount can be calculated using various models. The simplest and most widely applied model uses the Sauerbrey equation, which relates the change in frequency, Δ*f*, to the change in adsorbed mass per unit area, *m*.
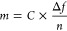
where *C* is a sensitivity constant −0.177 mg m^–2^ Hz^–1^, Δ*f* is the change in resonant frequency (Hz), and *n* is the overtone number. The third harmonic (*n* = 3) was used to calculate the adsorbed amount to avoid experimental artifacts associated with the fundamental harmonic. The Sauerbrey equation has been shown to be applicable when the Δ*D*_*n*_/Δ*f*_*n*_ ratio is greater than 0.1 × 10^–7^ Hz^–1^.^60,61^ All adsorbed masses presented herein are validated using this ratio.

#### Scanning Electron Microscopy

Scanning electron microscopy (SEM) images were acquired using an FEI Inspect F field emission scanning electron microscope operating at an acceleration voltage of 10−20 kV. All samples were prepared by drying dilute aqueous nanoparticle dispersions (0.1% w/w) onto silicon wafers at 25 °C. The sample-loaded silicon wafers were mounted on aluminum stubs using adhesive carbon tabs. Silver paint was applied to two edges of the mounted silicon wafers before sputter-coating with a gold overlayer to prevent charge build-up. Surface coverages (%) for the adsorbed nanoparticles were assessed using *ImageJ* software and were averaged across multiple images recorded for each sample.

### Synthesis Protocols

#### Preparation of the PGMA_51_ Precursor

GMA (78.144 g, 488 mmol), CPDB RAFT agent (1.9630 g, 8.873 mmol; target DP = 55), and ACVA (0.4970 g, 1.774 mmol; CPDB/ACVA molar ratio = 5.0) were weighed into a 500 mL round-bottom flask and degassed with a stream of nitrogen gas for 15 min. Ethanol (148 mL) was deoxygenated separately with nitrogen for 30 min prior to its addition to the other reagents. The reaction solution was stirred and degassed in an ice bath for a further 30 min before placing in an oil bath set at 70 °C. The polymerization was allowed to proceed for 150 min, resulting in a GMA conversion of 68% as judged by ^1^H NMR spectroscopy. The crude homopolymer was purified by precipitation into a ten-fold excess of DCM from methanol. This purification process was repeated twice to give a purified PGMA precursor (53.14 g, <1% residual GMA monomer; see Figure S1). Its mean DP was calculated to be 51 by end-group analysis using ^1^H NMR spectroscopy. DMF GPC analysis indicated an *M*_n_ of 13,200 g mol^–1^ and an *M*_w_/*M*_n_ of 1.23 (vs a series of near-monodisperse PMMA calibration standards).

#### RAFT Aqueous Emulsion Polymerization of Benzyl Methacrylate

A typical protocol for the synthesis of PGMA_51_–PBzMA_200_ diblock copolymer nanoparticles was conducted as follows: PGMA_51_ precursor (0.30 g, 35.76 μmol), BzMA (1.28 g, 7.273 mmol), ACVA (2.5 mg, 8.939 μmol; CTA/ACVA molar ratio = 4.0), and water (14.3 g, targeting 10% w/w solids) were weighed into a 50 mL round-bottom flask and purged with nitrogen for 30 min at 20 °C. Then the flask was immersed in an oil bath set at 70 °C for 24 h. The reaction mixture was stirred with the aid of a magnetic flea, and the resulting diblock copolymer was analyzed by DMF GPC (*M*_n_ = 27,400 g mol^–1^, *M*_w_/*M*_n_ = 1.35 vs PMMA standards). ^1^H NMR spectroscopy analysis of a freeze-dried sample dissolved in DMSO-*d*_6_ indicated less than 1% residual BzMA monomer. DLS studies of a 0.20% (w/w) copolymer dispersion indicated an intensity-average particle diameter of 64 nm (DLS polydispersity, PDI = 0.07).

#### Silicon Wafer Cleaning

Silicon (100) wafers were cut into small pieces (∼1 × 2 cm) before being cleaned by UV–ozone treatment for 60 min at 103 Pa using a BioForce Nanosciences ProCleaner. Wafers were then immersed in 2 M NaOH solution for 30 s, rinsed with Milli-Q water, and dried under compressed air prior to further UV–ozone treatment for 30 min.

#### Preparation of Initiator-Functionalized Silicon Wafers and QCM Sensors

Cleaned wafers and/or QCM sensors were placed in a Petri dish with a vial containing ∼400 μL of APTES within a desiccator. A vacuum of 5 mbar was achieved within 5 min. Then the tap was closed, and the wafers were maintained under vacuum for 30 min at 20 °C. Substrates were then annealed in air for 30 min at either 110 °C (wafers) or 70 °C (QCM sensors).

After cooling to 20 °C, these APTES-modified substrates were immersed in anhydrous DCM before triethylamine and BiBB were sequentially added to the solution. The final concentrations of triethylamine and BiBB were both 0.2 mM (triethylamine/BiBB molar ratio = 1:1). The surface was reacted for 60 min. Each wafer or QCM substrates were then rinsed with ethanol and Milli-Q water before being dried under a stream of compressed air.

#### PGEO5MA Brush Growth via SI-ARGET ATRP

PGEO5MA brushes were grown from initiator-functionalized silicon wafers and QCM sensors using a previously reported protocol.^62^ A polymerization mixture containing a 45% v/v aqueous solution of GEO5MA monomer, CuCl_2_ catalyst, and PMDETA ligand was prepared in deionized water at a molar ratio of 1000:1:10. Ascorbic acid (ascorbic acid/CuCl_2_ molar ratio = 4.0) was then dissolved in this aqueous mixture, which was stirred for 5 min at 20 °C. Either initiator-functionalized wafers and/or QCM sensors were immersed in this reaction solution, and the surface polymerization was allowed to proceed for 1 h. Substrates were removed and rinsed with copious amounts of ethanol and deionized water to quench the polymerization and then dried under a stream of compressed air.

#### Oxidation of a PGEO5MA Brush to Produce a PAGEO5MA Brush

As previously reported,^59,62^ wafers and/or QCM sensors were immersed in a 3.0 g dm^–3^ aqueous solution of NaIO_4_ for 30 min at 20 °C, washed extensively with ethanol and water, and then dried under a stream of compressed air.

#### Adsorption of PGMA_51_–PBzMA_200_ Nanoparticles onto a PAGEO5MA Brush

An aqueous dispersion of PGMA_51_–PBzMA_200_ nanoparticles was diluted to 1.0% w/w (∼3 mL) with water and adjusted to pH 4, 7, or 10 using dilute HCl or NaOH as required. Brush-functionalized wafers (or bare silicon wafers in control experiments) were then immersed in these aqueous dispersions. Nanoparticle adsorption was allowed to proceed for 18 h at 22 °C prior to thorough rinsing with water (10 mL, at the same solution pH as that of the nanoparticle dispersion) and drying under a stream of compressed air.

## Results and Discussion

**Scheme 1:**
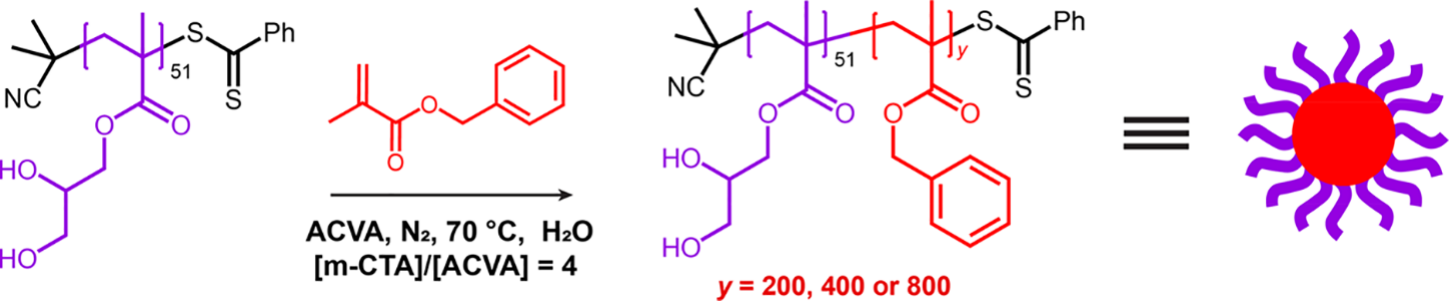
Synthesis of PGMA_51_–PBzMA_*x*_ Diblock Copolymer Nanoparticles via RAFT Aqueous Emulsion Polymerization of BzMA to Afford Spherical Nanoparticles. Increasing the Target DP of the PBzMA Block (*y*) Leads to a Systematic Increase in the Mean Nanoparticle Diameter

A series of PGMA_51_–PBzMA_*y*_ diblock copolymer nanoparticles (*y* = 200, 400 or 800) were prepared via RAFT aqueous emulsion polymerization of BzMA using a PGMA_51_ precursor (Figure S1), as shown in Scheme 1. The PGMA steric stabilizer chains bear *cis*-diol functionality, which was exploited by Cunningham et al. to modulate their surface adsorption onto a patterned planar surface via dynamic covalent chemistry.^52^ In the PISA literature, it is well-established that increasing the target DP of the core-forming block for a given steric stabilizer DP leads to a systematic increase in nanoparticle diameter.^46,63−67^^1^H NMR studies revealed that almost complete BzMA conversion (>99% conversion) was achieved within 24 h (Figure S2) while GPC analysis indicated efficient chain extension in each case (Figure S3), confirming the synthesis of well-defined diblock copolymers.

**Figure 1:**
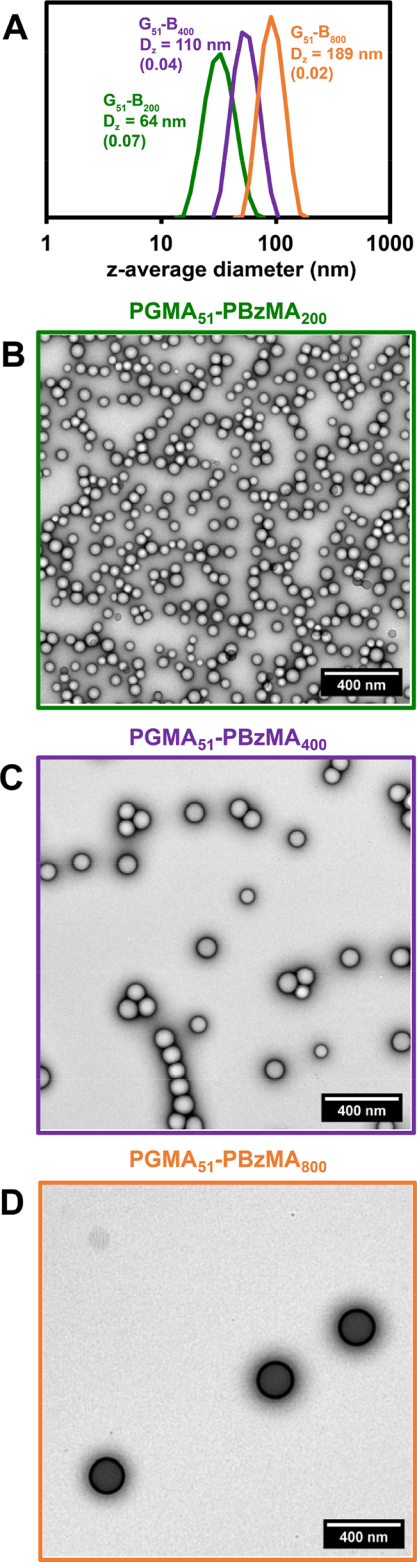
PGMA_51_–PBzMA_*y*_ diblock copolymer nanoparticles (*y* = 200, 400 or 800) prepared via RAFT aqueous emulsion polymerization of BzMA at 70 °C: (a) DLS particle size distributions (where “G” denotes PGMA and “B” denotes PBzMA) and (b−d) representative TEM images recorded for the three types of nanoparticles, illustrating their well-defined spherical morphology.

DLS studies indicated that the z-average diameter of the nanoparticles scaled proportionally with that of the target PBzMA DP (Figure 1a). TEM analysis confirmed a well-defined spherical morphology for each of the PGMA_51_–PBzMA_*y*_ nanoparticles, see Figure 1b−d. TEM and DLS particle size data are summarized in Table S1. It is perhaps worth mentioning that the PBzMA DPs targeted in this study were selected to ensure that the nanoparticles were sufficiently large to be readily discernible by SEM (see below).

Aqueous electrophoresis measurements (Figure S4) indicated that the nanoparticles were essentially neutral from pH 4 to pH 8 (zeta potentials ranged from −1 to −5 mV). Weakly anionic character was observed at pH 9–10 (zeta potential ∼ −10 mV). This is most likely the result of using an ACVA initiator for the synthesis of the PGMA precursor. For RAFT polymerization, it is well-known that a minor fraction of chains become capped with initiator fragments, rather than the R group originating from the RAFT agent.^68^ In the case of ACVA, this introduces a small number of carboxylic acid groups, which would account for the weakly anionic character observed at higher pH. One reviewer of this article has suggested that partial hydrolysis of the PGMA steric stabilizer chains might occur during the aqueous electrophoresis measurements. Such a side reaction would introduce methacrylic acid residues, which could contribute to the weakly negative zeta potentials indicated in Figure S4.

**Scheme 2:**
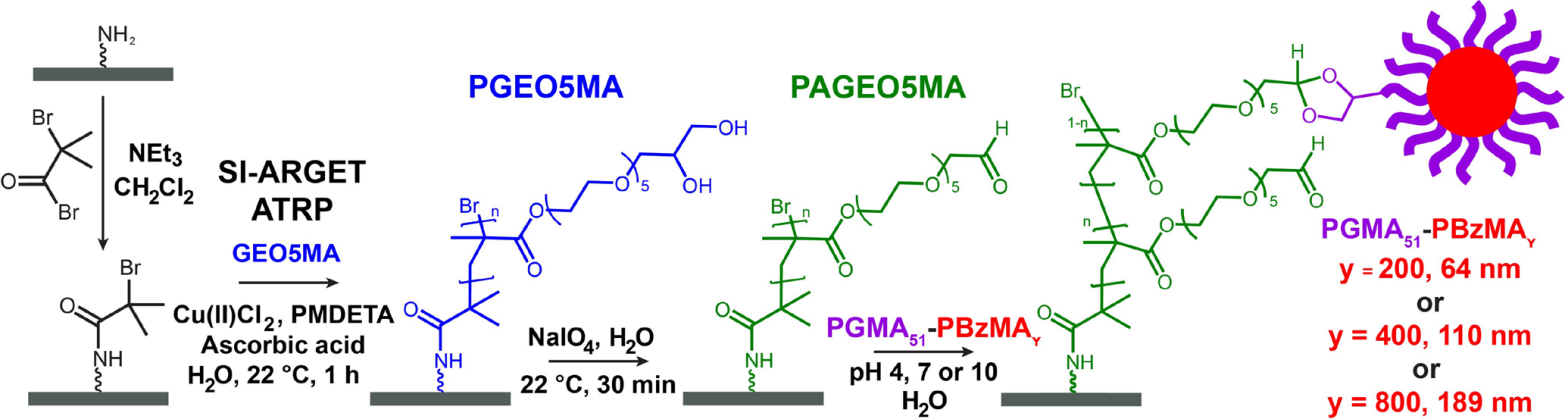
Growth of a PGEO5MA Brush from a Planar Silicon Wafer via SI-ARGET ATRP Followed by Selective Oxidation of the Pendent *cis*-Diol Groups with NaIO_4_ to Produce an Aldehyde-Functionalized PAGEO5MA Brush. Schematic Representation of the Attempted Adsorption of PGMA_51_–PBzMA_*y*_ Nanoparticles (*y* = 200, 400 or 800; See Scheme 1) onto the Aldehyde-Functionalized Brush via Acetal Bond Formation

PGEO5MA brushes were prepared using our previously reported SI-ARGET ATRP protocol (Scheme 2).^62^ Spectroscopic ellipsometry was used to determine dry brush thicknesses (∼35 nm within 60 min of surface polymerization), with smooth brush surfaces and satisfactory data fits being achieved in each case. Each PGEO5MA brush was then oxidized using a 3.0 g dm^–3^ aqueous solution of NaIO_4_. Subsequent ellipsometry studies of the dried brush revealed an approximate 7% reduction in the dry brush thickness: this is consistent with the lower repeat unit mass expected after the loss of formaldehyde associated with selective oxidation.^62,69^

Non-ionic and zwitterionic hydrophilic polymer brushes are well-known for their ability to minimize biofouling.^70,71^ On close approach to a well-solvated brush, proteins (and other biomacromolecules) typically experience a strong repulsive interaction owing to an increase in osmotic pressure.^72,73^ The magnitude of this force depends on the inter-separation distance, brush grafting density,^72^ and particle size,^74,75^ as well as the precise geometry.^76^

**Scheme 3:**
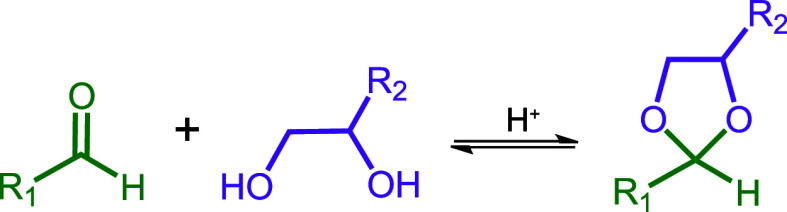
Acid-Catalyzed Acetal Bond Formation When Reacting an Aldehyde with a *cis*-Diol Compound

If nanoparticle adsorption is desired, then an attractive interaction force between the brush and the nanoparticles must be introduced to overcome the repulsive force. Recently, we exploited dynamic covalent chemistry (more specifically, imine bond formation) to adsorb aldehyde-functional nanoparticles onto amine-functional polymer brushes.^59^ Herein we examine whether the formation of acetal bonds between *cis*-diol-functional PGMA_51_–PBzMA_*y*_ nanoparticles and a hydrophilic aldehyde-functional polymer brush is sufficient to promote adsorption. It is well-known that acetal bond formation is (i) acid-catalyzed and (ii) more entropically favorable when using a diol rather than two separate alcohols.^77^ The general reaction for acetal bond formation between an aldehyde and a *cis*-diol compound is shown in Scheme 3.

**Figure 2:**
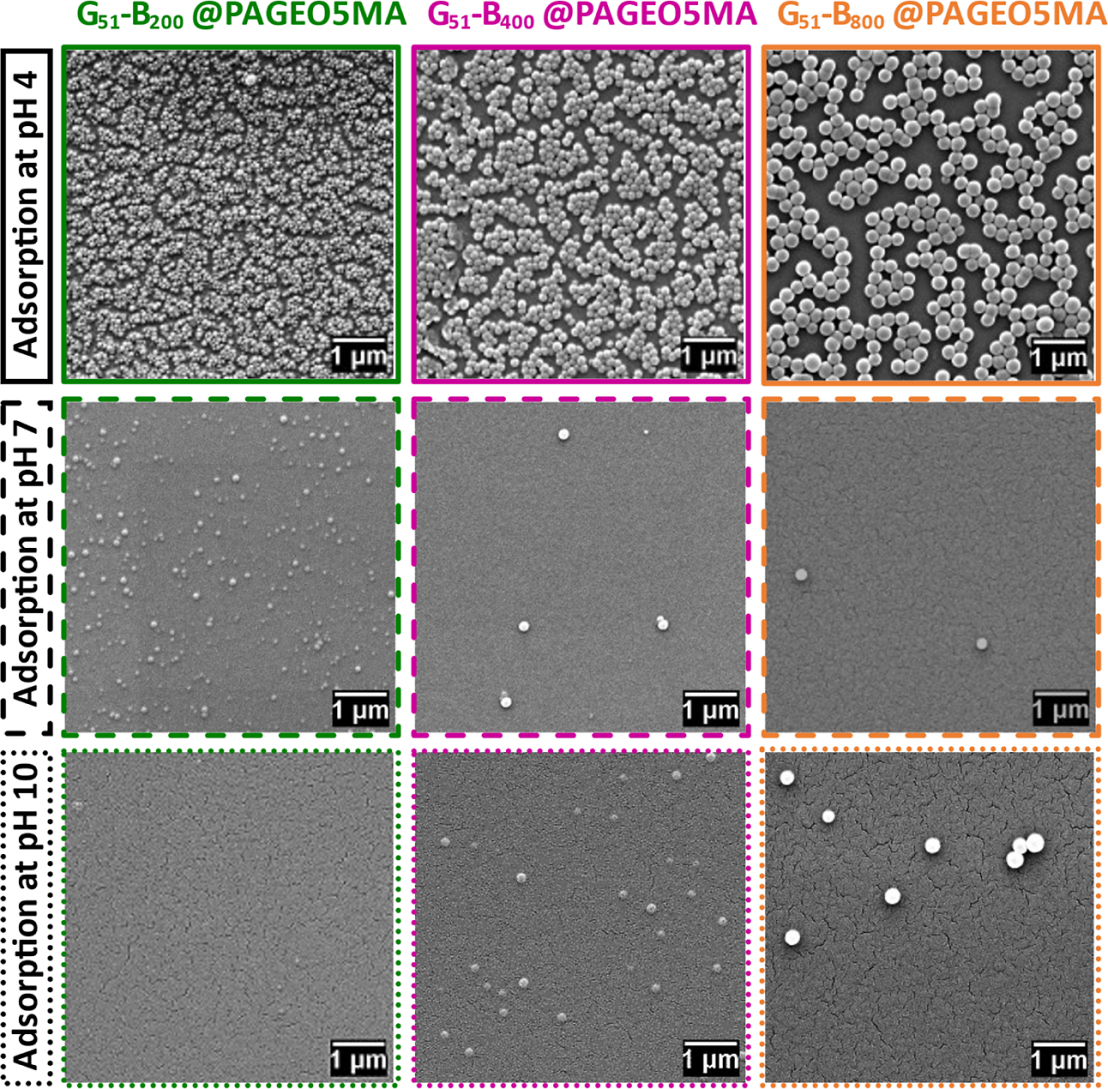
SEM images recorded for a series of PGMA_51_–PBzMA_*y*_ nanoparticles (where *y* = 200, 400, or 800) adsorbed onto PAGEO5MA brushes (∼32 nm dry brush thickness) grown from planar silicon wafers. Nanoparticle adsorption experiments were conducted at 25 °C using aqueous dispersions adjusted to pH 4, 7, or 10.

PAGEO5MA brushes were immersed in aqueous dispersions containing the three different types of nanoparticles at pH 4, 7, or 10, followed by rinsing with copious amounts of water at the same pH. The extent of nanoparticle adsorption was assessed by SEM (Figure 2). Clearly, extensive nanoparticle adsorption occurs at pH 4, with surface coverages (estimated by digital image analysis using *ImageJ* software) ranging from 50 to 61%. The apparent clustering at these very high surface coverages has been observed previously for similar nanoparticles adsorbed at planar interfaces.^14,38^ In striking contrast, nanoparticle adsorption is much weaker at both pH 7 and pH 10 (surface coverage < 1−2% in each case). A summary of the surface coverage data is provided in Table S2. The remarkable enhancement in nanoparticle adsorption observed under mildly acidic conditions illustrates the importance of pH modulation in facilitating acetal bond formation. Moreover, these observations are consistent with prior small-molecule studies.^78^

QCM is widely used for the construction of various types of biosensors^17−20^ and can also be used to characterize the viscoelastic properties of thin polymer films such as polymer brushes.^79−82^ We have recently employed QCM to quantify the extent of adsorption of either diblock copolymer nanoparticles or nanosized oil droplets onto model soft substrates^14,15,83^ and for the conjugation of a globular protein to a PAGEO5MA brush via Schiff base chemistry.^62^ Herein we employ QCM to quantify the adsorption of PGMA_51_–PBzMA_*y*_ nanoparticles (*y* = 200, 400, or 800) onto PAGEO5MA brushes by determining the adsorbed mass per unit area (Γ, mg m^–2^).^84,85^

**Figure 3:**
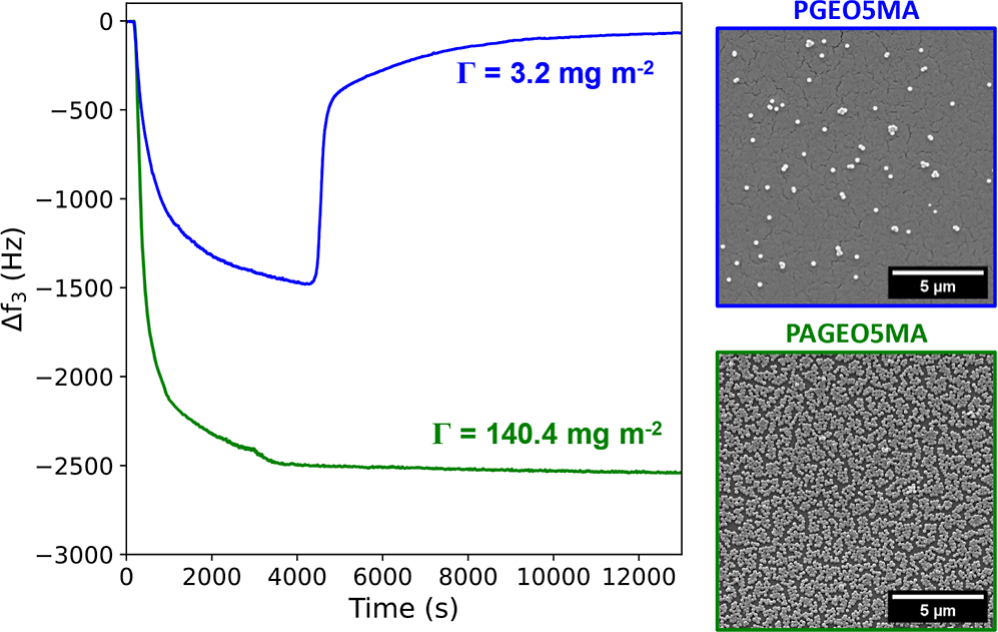
QCM experiments and SEM studies conducted at 25 °C for the adsorption of PGMA_51_–PBzMA_800_ nanoparticles onto either PAGEO5MA or PGEO5MA brushes (≈32 nm dry brush thickness) grown from either a silica QCM sensor or a planar silicon wafer (SEM). Adsorbed amounts are calculated from QCM experiments using the Sauerbrey equation.

With QCM, we first demonstrate that the aldehyde groups within the hydrophilic brush chains are essential for strong nanoparticle adsorption. PGEO5MA and PAGEO5MA brushes were grown from separate QCM sensors before being exposed in turn to the same 1.0% w/w dispersion of PGMA_51_–PBzMA_800_ nanoparticles at pH 4. A dramatic reduction in frequency was observed for the aldehyde-functional brush, which indicates strong nanoparticle adsorption at 25 °C; see Figure 3. Importantly, the change in frequency remains constant after rinsing with water at pH 4, indicating irreversible nanoparticle adsorption. In contrast, only a moderate reduction in frequency was observed for the hydroxy-functional PGEO5MA brush at the same temperature. Moreover, this frequency returns to close to its original value after rinsing with water at pH 4, indicating that most of the initially adsorbed nanoparticles are only weakly bound and can be readily washed off the brush surface. These findings are corroborated by the SEM images shown in Figure 3.

The adsorbed amount, Γ, calculated from the QCM data for nanoparticle adsorption on both types of brushes is indicated within Figure 3. The striking difference in the extent of adsorption for *cis*-diol-functional polymer nanoparticles on the *cis*-diol- and aldehyde-functional polymer brushes underlines the importance of acetal bond formation in promoting nanoparticle adsorption. In a separate control experiment, SEM studies confirmed that nanoparticle adsorption onto a bare silicon wafer (i.e., no brush layer) was negligible (Figure S6).

**Figure 4:**
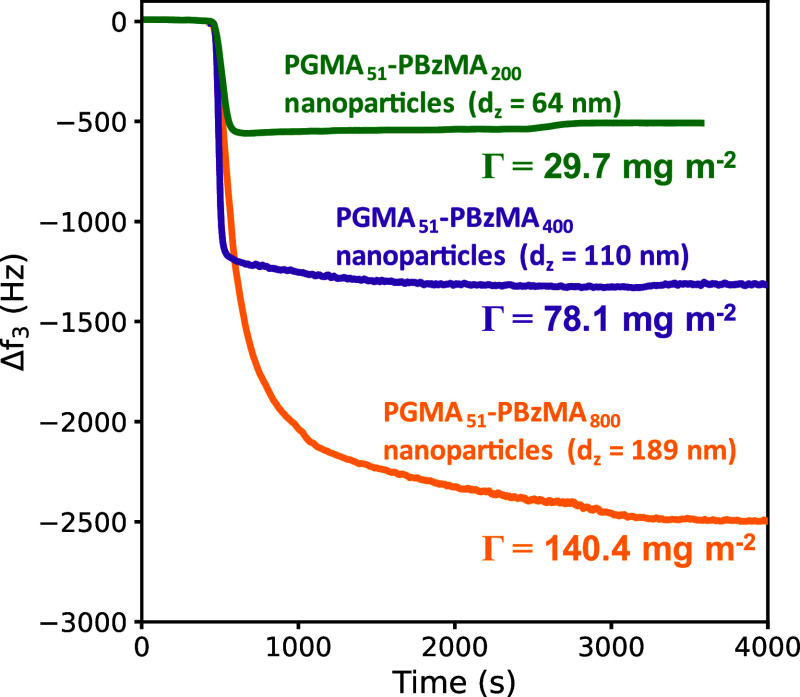
QCM experiments conducted at 25 °C for the adsorption of PGMA_51_–PBzMA_*y*_ nanoparticles [for *y* = 200 (upper curve), *y* = 400 (middle curve), or *y* = 800 (lower curve)] onto PAGEO5MA brushes at pH 4.

QCM was also employed to quantify the adsorbed mass of the three types of nanoparticles on the PAGEO5MA brush layer (Figure 4). According to Figure 2, the mean nanoparticle surface coverage is approximately constant regardless of the particle size. However, a larger reduction in frequency was observed with increasing nanoparticle diameter. This is physically reasonable as mass scales with the cube of the nanoparticle radius, whereas surface area scales only as the square of the nanoparticle radius.

**Figure 5:**
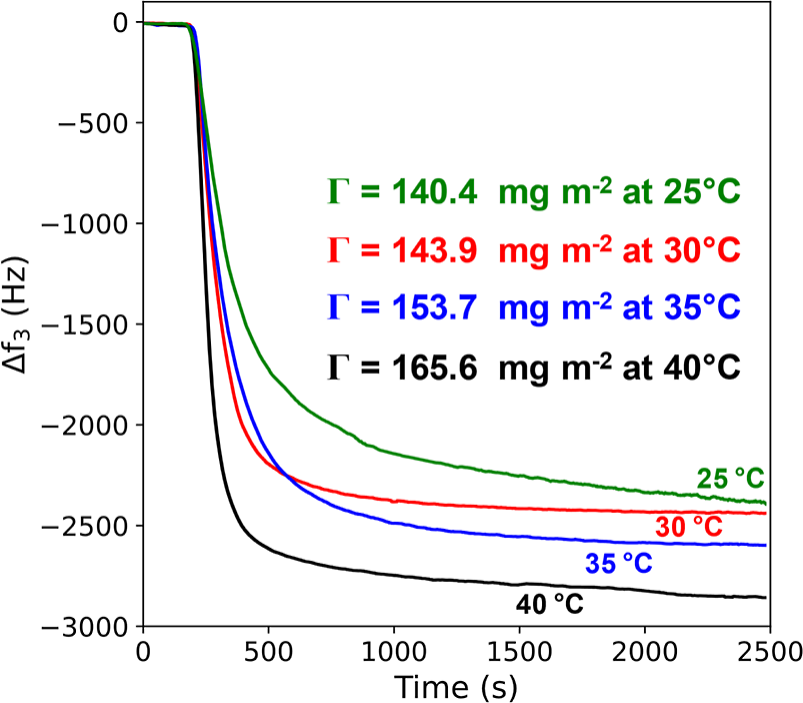
Temperature dependence of the adsorbed amount for PGMA_51_–PBzMA_800_ nanoparticles bound to a PAGEO5MA brush at pH 4 for the 25–40 °C interval as indicated by QCM studies. The adsorbed amount is arbitrarily determined after 2500 s (25 min) in each case. Greater adsorption at higher temperature provides strong evidence for chemical adsorption of the nanoparticles via acetal bond formation.

Finally, we sought to demonstrate that the adsorption of *cis*-diol-functionalized nanoparticles onto aldehyde-functional brushes really does involve chemical adsorption (i.e., acetal bond formation), rather than merely strong physical adsorption. In principle, these two modes of adsorption can be distinguished by performing temperature-dependent adsorption studies.^15,86^ Thus, in the case of chemical adsorption, the adsorbed amount should *increase* with temperature, since this favors the formation of additional acetal bonds.^87,88^ In contrast, merely physical adsorption should be characterized by a *reduction* in the adsorbed amount. Accordingly, the adsorption of PGMA_51_–PBzMA_800_ nanoparticles onto a PAGEO5MA brush was studied by QCM from 25 to 40 °C, see Figure 5. Clearly, the adsorbed amount increases significantly at higher temperatures, which is consistent with the proposed formation of acetal bonds between the nanoparticles and the brush chains.

## Conclusions

Three examples of sterically stabilized diblock copolymer nanoparticles bearing *cis*-diol functionality were prepared using an efficient aqueous PISA formulation. A well-defined spherical morphology was observed in each case, and the DLS z-average particle diameter was readily adjusted from 64 to 189 nm simply by varying the target DP for the core-forming poly(benzyl methacrylate) block.

In mildly acidic aqueous solution, such hydroxy-functional nanoparticles react readily with an aldehyde-functional hydrophilic brush, with QCM and SEM studies indicating relatively high adsorbed amounts of 140 mg m^–2^ and surface coverages of up to 61%, respectively. However, only minimal nanoparticle adsorption occurs in either neutral or alkaline solution, indicating that exquisite control over the extent of adsorption can be achieved via pH modulation. Finally, temperature-dependent QCM studies confirm that significantly higher adsorbed amounts are obtained at higher temperatures, which provides strong evidence for chemical adsorption via acetal bond formation. This model system provides an exemplar for the interaction of reactive sterically stabilized nanoparticles with soft substrates (e.g., hydrophilic polymer brushes).

## Supporting Information Available

Assigned ^1^H NMR spectra for the PGMA_51_ precursor and PGMA_51_–PBzMA_200_; DMF GPC data for PGMA_51_, PGMA_51_–PBzMA_200_, PGMA_51_–PBzMA_400_, and PGMA_51_–PBzMA_800_; summary of DLS and TEM particle size data; zeta potential vs pH curves obtained for 1.0% w/w aqueous dispersions of PGMA_51_–PBzMA_200_, PGMA_51_–PBzMA_400_, and PGMA_51_–PBzMA_800_ nanoparticles; summary of surface coverages calculated from SEM images after nanoparticle adsorption at pH 4, 7, or 10 onto a PAGEO5MA brush; SEM images recorded after the attempted adsorption of PGMA_51_–PBzMA_800_ nanoparticles at pH 4, 7, or 10 onto a PGEO5MA brush; and SEM images recorded after the attempted adsorption of PGMA_51_–PBzMA_800_ nanoparticles at pH 4, 7, or 10 onto a planar silicon wafer (PDF)
