# Supplementary material for: Controlling Adsorption of Diblock Copolymer Nanoparticles onto an Aldehyde-Functionalized Hydrophilic Polymer Brush via pH Modulation
Source: Langmuir. 2024 Feb 6;40(7):3667–76. doi: 10.1021/acs.langmuir.3c03392 (PMC10883040; doi:10.1021/acs.langmuir.3c03392)
Supplement: Supplementary file 1 — la3c03392_si_001.pdf [file la3c03392_si_001.pdf]

Supporting Information for:

Controlling Adsorption of Diblock Copolymer Nanoparticles onto an Aldehyde-functionalized Hydrophilic Polymer Brush via pH Modulation

Samuel Astier[a], Edwin C. Johnson[a,*], Oleta Norvilaite[a], Spyridon Varlas[a], Emma E. Brotherton[a], George Sanderson[b], Graham J. Leggett[a] and Steven P. Armes[a,*]

a. Dainton Building, Department of Chemistry, The University of Sheffield, Brook Hill, Sheffield, South Yorkshire, S3 7HF, UK.

b. GEO Specialty Chemicals, Hythe, Southampton, Hampshire SO45 3ZG, UK.

* Corresponding authors (e.c.johnson@sheffield.ac.uk or s.p.armes@sheffield.ac.uk)

Summary of Contents

Figure S1. Assigned ^{1}H NMR spectrum for the $PGMA_{51}$ precursor.

Figure S2. Assigned ^{1}H NMR spectra (d_7-DMF) recorded for $PGMA_{51}$-$PBzMA_{200}$ reaction mixtures, purified $PGMA_{51}$-$PBzMA_{200}$ and BzMA monomer.

Figure S3. DMF GPC curves recorded for $PGMA_{51}$, $PGMA_{51}$-$PBzMA_{200}$, $PGMA_{51}$-$PBzMA_{400}$ and $PGMA_{51}$-$PBzMA_{800}$.

Table S1. Summary of particle size data obtained from DLS measurements and TEM images for the three types of nanoparticles used in this study. [TEM diameters were estimated by analysis of approximately 100 nanoparticles].

Figure S4. Zeta potential vs. pH curves obtained for 1.0% w/w aqueous dispersions of $PGMA_{51}$-$PBzMA_{200}$, $PGMA_{51}$-$PBzMA_{400}$, and $PGMA_{51}$-$PBzMA_{800}$ nanoparticles in 1 mM KCl.

Table S2. Summary of surface coverages calculated from the digital image analysis of SEM images recorded after the adsorption of $PGMA_{51}$-$PBzMA_{200}$, $PGMA_{51}$-$PBzMA_{400}$, or $PGMA_{51}$-$PBzMA_{800}$ nanoparticles at pH 4, 7 or 10 onto a PAGEO5MA brush grown from a planar silicon wafer.

Figure S5. SEM images recorded after the attempted adsorption of $PGMA_{51}$-$PBzMA_{800}$ nanoparticles at pH 4, 7 or 10 onto a PGEO5MA brush grown from a planar silicon wafer (control experiments).

Figure S6. SEM images recorded after the attempted adsorption of $PGMA_{51}$-$PBzMA_{800}$ nanoparticles at pH 4, 7 or 10 onto a planar silicon wafer (control experiments).

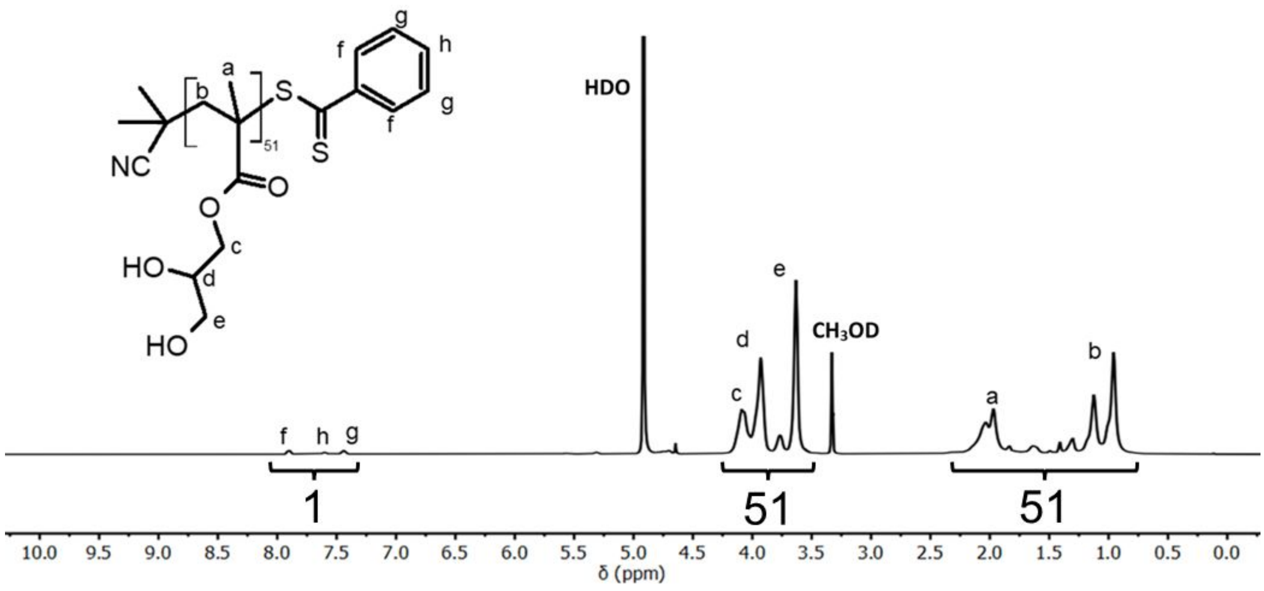

Figure S1 Assigned ^{1}H NMR spectrum (CD_3OD) for the $PGMA_{51}$ precursor used for the nanoparticle syntheses described in this study. [N.B. Hydroxyl protons are not visible owing to H/D exchange in this protic solvent].

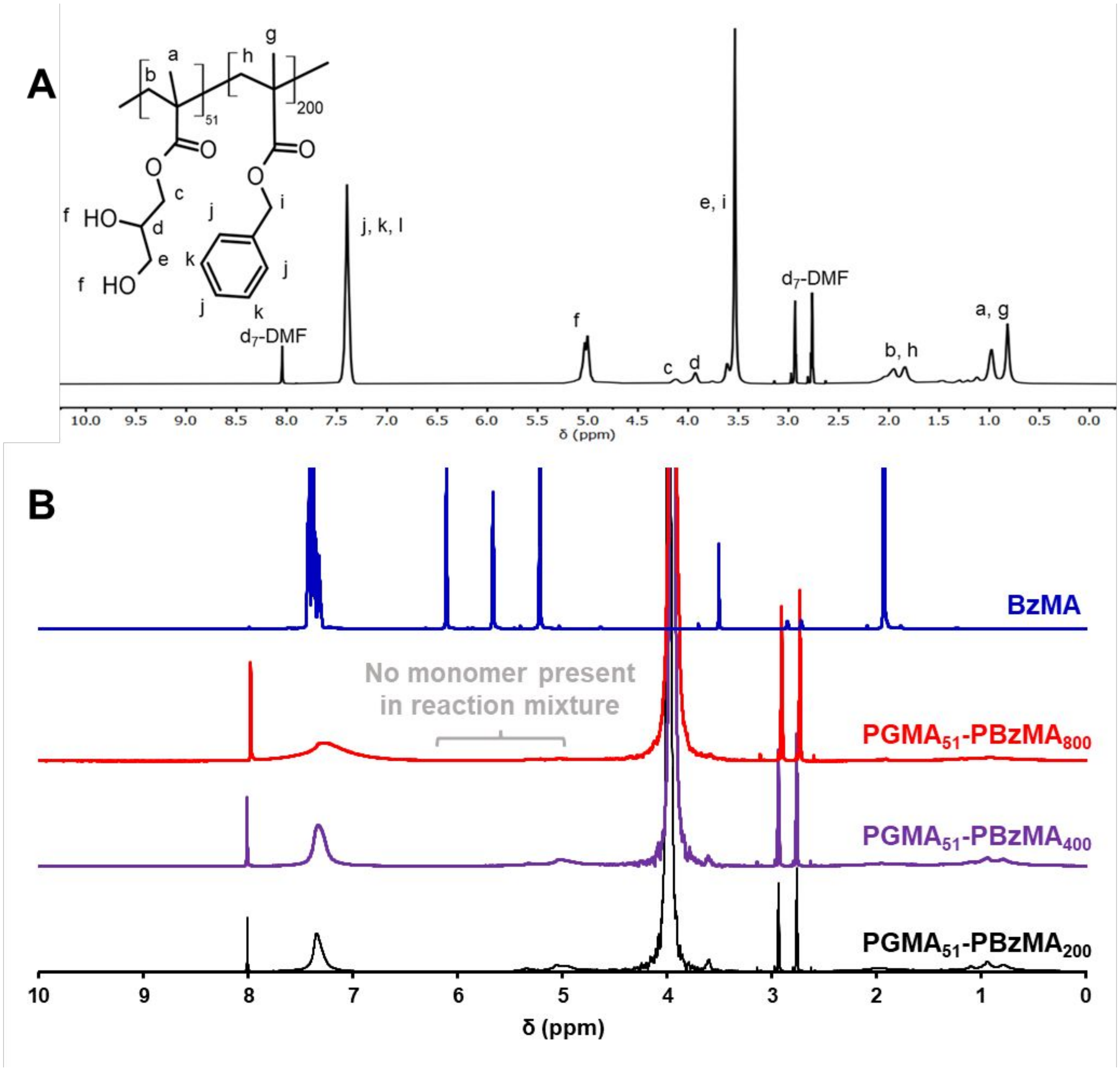

Figure S2 (A) Assigned ^{1}H NMR spectrum (d_7-DMF) for $PGMA_{51}$-$PBzMA_{200}$ chains. (B) ^{1}H NMR spectrum (d_7-DMF) for BzMA monomer (blue spectrum) and the three reaction mixtures (for $PGMA_{51}$-$PBzMA_x$, where x = 200, 400 or 800). The latter three spectra contain no evidence for residual BzMA monomer, indicating very high final BzMA conversions (>99%).

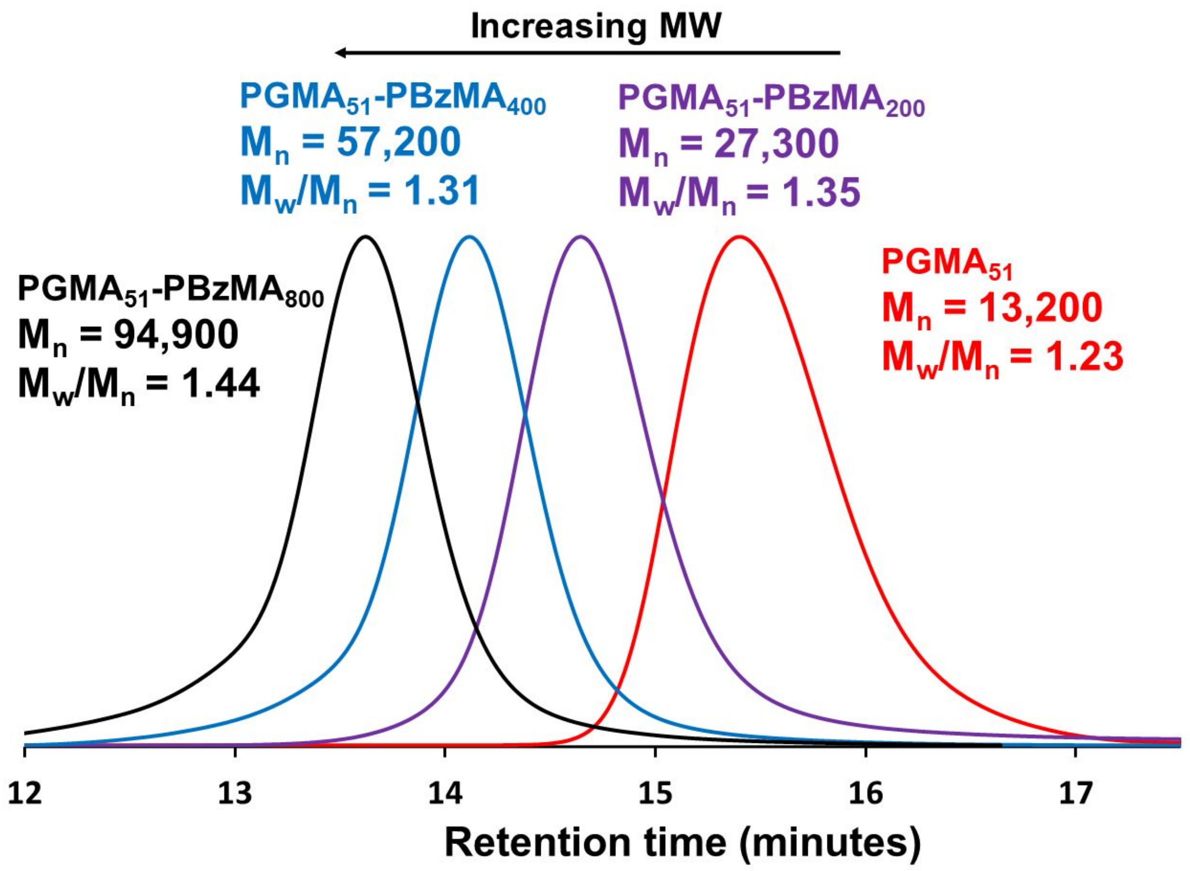

Figure S3. DMF GPC curves recorded for $PGMA_{51}$, $PGMA_{51}$-$PBzMA_{200}$, $PGMA_{51}$-$PBzMA_{400}$ and $PGMA_{51}$-$PBzMA_{800}$. Molecular weight data are expressed relative to a series of near-monodisperse poly(methyl methacrylate) standards.

Copolymer composition	DLS z-average diameter (nm)	TEM number-average diameter (nm)
$PGMA_{51}$-$PBzMA_{200}$	64	54
$PGMA_{51}$-$PBzMA_{400}$	110	86
$PGMA_{51}$-$PBzMA_{800}$	189	156

Table S1. Summary of particle size data obtained from DLS measurements and TEM images for the three types of nanoparticles used in this study. [TEM diameters were estimated by analysis of approximately 100 nanoparticles].

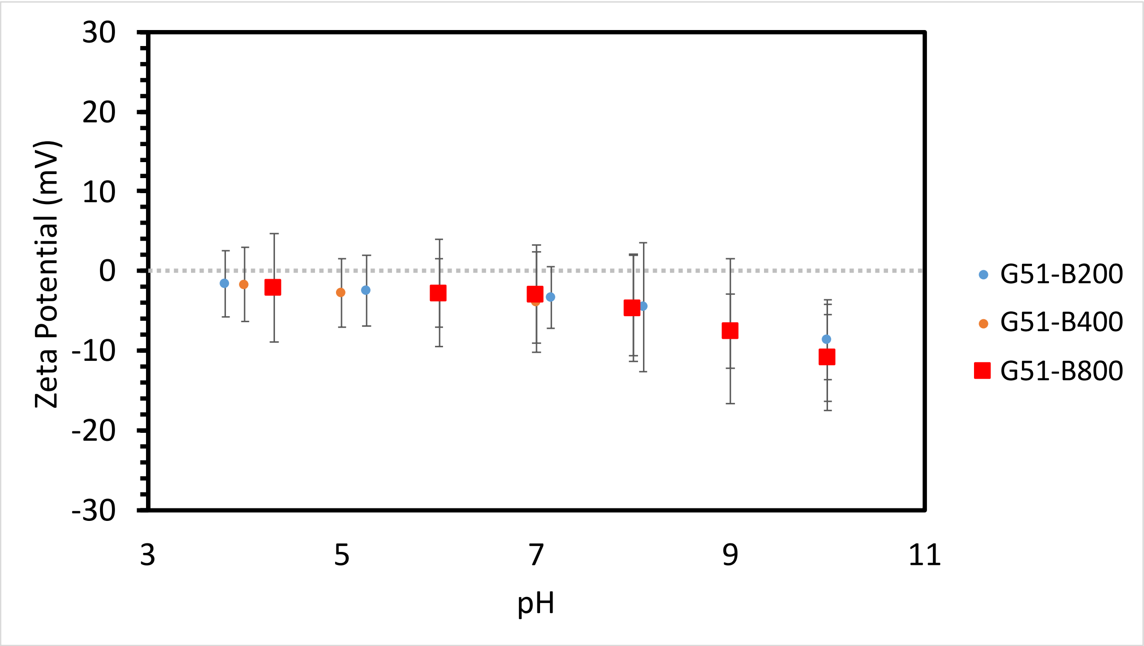

Figure S4. Zeta potential vs. pH curves obtained for 1.0% w/w aqueous dispersions of $PGMA_{51}$-$PBzMA_{200}$ (G_{51}-B_{200}), $PGMA_{51}$-$PBzMA_{400}$ (G_{51}-B_{400}), and $PGMA_{51}$-$PBzMA_{800}$ (G_{51}-B_{800}) nanoparticles in 1 mM KCl.

Solution pH	G_{51}-B_{200}	G_{51}-B_{400}	G_{51}-B_{800}
pH 4	0.50	0.61	0.61
pH 7	0.01	0.01	0.02
pH 10	0	0.01	0.01

Table S2. Summary of surface coverages calculated from the digital image analysis of SEM images recorded after the adsorption of $PGMA_{51}$-$PBzMA_{200}$, $PGMA_{51}$-$PBzMA_{400}$, or $PGMA_{51}$-$PBzMA_{800}$ nanoparticles at pH 4, 7 or 10 onto a PAGEO5MA brush grown from a planar silicon wafer.

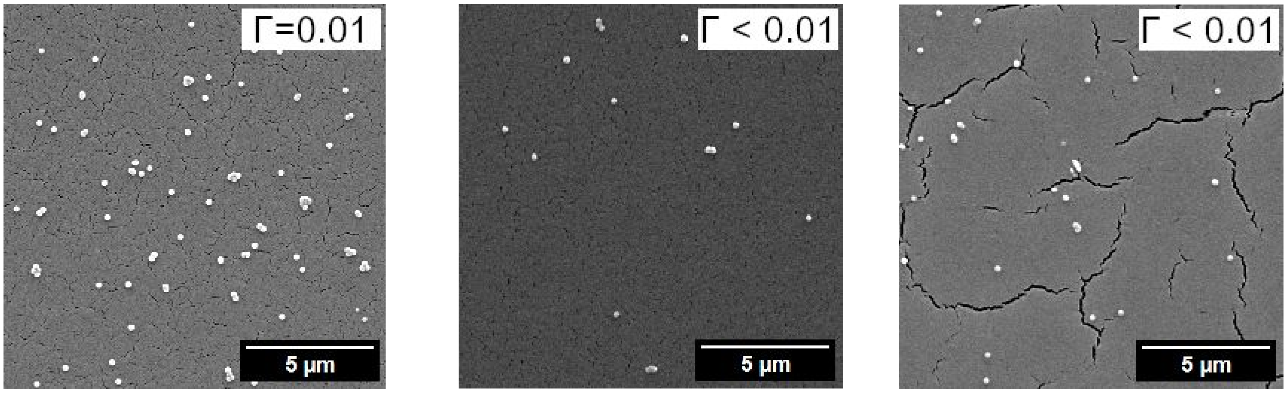

Figure S5. SEM images recorded after the attempted adsorption of $PGMA_{51}$-$PBzMA_{800}$ nanoparticles at pH 4, 7 or 10 onto a PGEO5MA brush grown from a planar silicon wafer (control experiments).

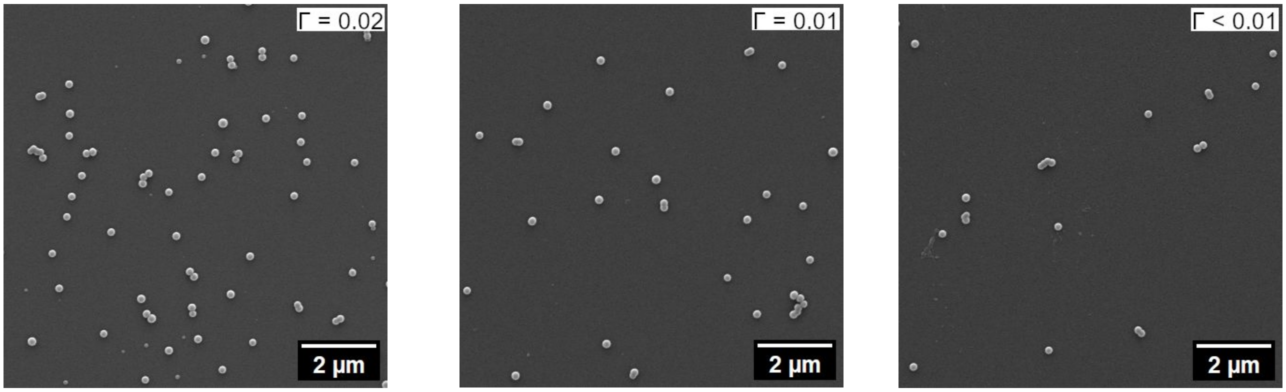

Figure S6. SEM images recorded after the attempted adsorption of $PGMA_{51}$-$PBzMA_{800}$ nanoparticles at pH 4, 7 or 10 onto a planar silicon wafer (control experiments).
